# Evaluation and Comparison of Manual and Mechanical Endodontic Instrumentation Completed by Undergraduate Dental Students on Endodontic Blocks

**DOI:** 10.3390/dj12110363

**Published:** 2024-11-14

**Authors:** António Ginjeira, Abayomi O. Baruwa, Karla Baumotte

**Affiliations:** 1 Faculdade de Medicina Dentária, Universidade de Lisboa, 1600-277 Lisboa, Portugal; aginjeira@campus.ul.pt (A.G.); karla.corte-real@campus.ul.pt (K.B.); 2 Grupo de Investigação em Bioquimica e Biologia Oral (GIBBO), Unidade de Investigação em Ciências Orais e Biomédicas (UICOB), 1600-277 Lisboa, Portugal

**Keywords:** endodontics, dental students, dental education, nickel–titanium, stainless steel

## Abstract

**Background:** The shaping of root canal space was completed using manual stainless steel files in earlier decades and with the advent of mechanical nickel–titanium (NiTi) instruments, there is potential for more efficient root canal preparation. Despite the advantages of NiTi instruments, their adoption in undergraduate dental education remains limited. The aim of this study was to evaluate three root canal instrumentation techniques, manual instrumentation using stainless steel hand files, continuous rotation employing ProTaper Gold (PTG) files, and reciprocation with WaveOne Gold (WOG) files, on endodontic resin blocks to assess the quality of preparation and the time required for instrumentation. **Methods**: A total of 36 third-year dental students, all lacking prior experience in root canal procedures, were divided into six groups to prepare 108 resin endodontic blocks with each student preparing 3 blocks. Images were captured at the preoperative, intraoperative, and postoperative stages to facilitate comparisons and measurements of the prepared blocks to assess the degree of resin removal, apical deviation, and mid-cervical wear. Furthermore, questionnaires were distributed to assess the students’ experiences and satisfaction with the techniques. The Friedman test, Wilcoxon test with Bonferroni correction, and Kruskal–Wallis test with Mann–Whitney U test were used to analyse and compare techniques, with the level of significance set at *p* < 0.05. **Results**: Instrumentation with PTG exhibited significantly reduced apical deviation (0.073 ± 0.003) compared to both the WOG and manual instrumentations (*p* < 0.001). Significant differences in mid-cervical wear were observed only between PTG and the manual instrumentation. In terms of resin removal, the manual instrumentation displayed greater variability and was five times slower to complete the instrumentation. In total, 90% of students favoured mechanical instrumentation, with substantial preferences for them over manual techniques. **Conclusions**: Mechanical instrumentation techniques, notably with the PTG system, were significantly faster and more effective in preparation quality. This highlights the potential for the inclusion of mechanical instrumentation in undergraduate dental curricula.

## 1. Introduction

Effective endodontic treatment relies on the integral principles of cleaning, shaping, and three-dimensional obturation [1,2]. The cleaning and shaping process involves the complete removal of the root canal contents to achieve a continuous and uniformly conical shape, preserving the canal’s diameter as compact as possible [3]. Achieving the root canal shaping involves the use of various instruments, each designed to serve a distinct purpose in achieving an ideal final preparation. Proficiency with an instrument depends on the clinician’s experience and a thorough understanding of the instrument’s features and kinematics [3]. For several decades, mechanical root canal preparation was predominantly performed using hand stainless steel files until the advent of mechanical root canal preparation with nickel–titanium (NiTi) files. The merits of NiTi instruments for root canal shaping have been extensively described and supported by a substantial body of literature for their cutting efficiency and their ability to create conservative preparations [3,4,5]. However, the widespread adoption of NiTi instruments remains predominantly in the realm of experienced clinicians and postgraduate training, with limited integration into the undergraduate curricula of most dental schools [6]. 

Education regulation bodies in dentistry and associations such as the European Society of Endodontology (ESE) and the Association for Dental Education in Europe (ADEE) have consistently emphasized in various publications and guidelines that the undergraduate curriculum should encompass a comprehensive spectrum of theoretical, practical, pre-clinical, and clinical training [7]. The goal is to equip students with the skills necessary to perform non-complex endodontic procedures. The ESE’s recognition of the fact that today’s students will be the clinical practitioner of the future underscores the need to formulate technically rigorous educational programs that prepare them to deliver high-quality endodontic care [7,8]. In this context, it is imperative to incorporate innovative techniques and instruments that have demonstrated efficacy into undergraduate curricula. This proactive approach not only enriches the education of students but also augments their clinical skills, making them better prepared for the demands of modern dentistry [9,10].

Despite the validated advantages of NiTi rotary instruments, the traditional manual techniques involving stainless steel files continue to be a staple in the undergraduate training programs of many dental schools. The introduction of mechanical root canal preparation techniques in undergraduate clinics has encountered resistance, primarily due to concerns surrounding instrument breakage and the initial costs associated with procuring new equipment [10]. Nevertheless, there is an emerging trend toward introducing mechanical techniques to undergraduate students, which is substantiated by an increasing body of research that reports excellent outcomes when NiTi instruments are employed in canal preparation by these aspiring dental professionals [6,11,12,13,14,15,16]. 

However, there are limited references in the literature addressing questions regarding the influence of the training sequence on the learning curve and its impact on the quality of preparations, and it remains uncertain whether instructing students in the manual technique with stainless steel instruments before introducing mechanical methods is necessary. Therefore, the objective of this research was to evaluate three different root canal instrumentation techniques (manual using stainless steel hand files, continuous rotation with ProTaper Gold files, and reciprocation movement with WaveOne Gold files) on resin blocks, carried out by third-year undergraduate dental students without previous experience and conduct a comparative analysis of the quality of the root canal preparation, the time required for instrumentation, and preference for each system. The null hypothesis to be tested is that there are no differences in the instrumentation techniques used regarding the quality of preparation, timing, and satisfaction. 

## 2. Materials and Methods

A cluster of all third-year undergraduates (thirty-six students) from the Faculty of Dental Medicine, University of Lisbon (FMDUL) participated in this study. As a strict inclusion criterion, the students must have no prior experience with the materials and techniques to be assessed. Before the laboratory phase, the students received theoretical instruction covering the instruments and techniques. They were briefed on the composition, physical properties, and design of both NiTi and stainless steel instruments, as well as instructions for the specific techniques used in this research. Notably, participation in this study was entirely voluntary, and the results obtained did not impact their academic grades; hence, Ethics Committee approval was waived under the protocol no. CEBD202402.

To ensure an unbiased evaluation, the 36 students were randomly assigned to six groups based on a randomized, blinded numbering system that was concealed from the primary operator (KB) who assessed the images and data. Each group was assigned to instrument a total of 108 single-canal resin blocks (approximately 18 blocks per group) in a specific sequence using stainless steel (SS), ProTaper Gold (PTG), and WaveOne Gold (WOG). The instrumentation sequences were as follows: Group I: SS-PTG-WOG; Group II: SS-WOG-PTG; Group III: PTG-SS-WOG; Group IV: PTG-WOG-SS; Group V: WOG-SS-PTG; and Group VI: WOG-PTG-SS. Each participant had the opportunity to use each of the techniques on different days during the laboratory sessions, which were conducted over three consecutive weeks, with a one-week interval between each session. During the preparatory session, each student was provided with a kit tailored to the technique they were assigned to work on. These kits included the files required for the day’s work, a millimetre ruler, a 5 mL irrigation syringe (Luer Lock) with a 27-gauge side-vented needle, a container with alcohol, the resin block slated for preparation (ISO 15, Endo-Training-Bloc-S. 02 Taper; Dentsply-Maillefer, Ballaigues, Switzerland), and a script outlining the laboratory sequence. The resin blocks used in this study measured 16.5 mm in total length, had a taper of 0.02, and featured an apical foraminal diameter of 0.15 mm. The working length for the block preparation was fixed at 16.0 mm, irrespective of the technique employed. Prior to the preparations, the canals’ permeability was verified using a 10 K file. Each participant was equipped with a stopwatch to record the time taken for the entire canal preparation, including instrumentation, file cleaning, and irrigation. Timekeeping was paused during the switching of files for the manual preparation and preparation with the PTG system.

The manual preparation using stainless steel (SS) files from size 10, 15, 20, and 25 K files followed the training module at the FMDUL Endodontics Discipline, which entailed using SS hand files in a crown-to-apex method, with kinematics based on the balanced force principle. This method included three phases: enlarging the cervical and middle thirds, using Gates–Glidden drills, dilating the apical third, and establishing a taper along the entire canal length through programmed indentations. Canal irrigation with alcohol and permeability checks were performed with each file change. Regarding continuous rotation preparation with the PTG file (Dentsply Sirona-Maillefer, Ballaigues, Switzerland), the preparation adhered to the manufacturer’s guidelines and endomotor settings. The canal was initially irrigated with alcohol, and all files were introduced passively. Sequentially, various files (SX, S1, S2, F1, F2) were used. SX, S1, and S2 files were used in sweeping and penetrating movements, cutting dentin at the canal exit. In contrast, F1 and F2 files were used with penetration and exit movements, but without applying pressure on the canal walls. For the reciprocating motion, preparation was completed using the WOG primary file (Dentsply Sirona-Maillefer, Ballaigues, Switzerland) according to the manufacturer’s instructions, with files driven by a pre-programmed motor featuring reciprocating kinematics. Files were used in a pecking motion, involving small insertion and withdrawal strokes along the long axis of the instrument/canal, with a maximum amplitude of 3 mm. After every three pecking cycles, the file was removed, cleaned, and the canal was irrigated, with permeability checked using a 10 K file. To maintain consistency and enable effective comparisons among all techniques, the instrumentation was completed at a standardized apical diameter of size 25.

### 2.1. Imaging

**Figure 1:**
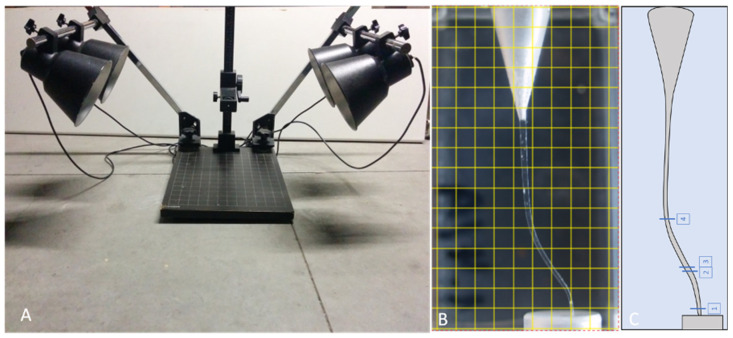
(**A**) Image capturing and reproduction table, (**B**) grid overlay, and (**C**) measuring points 1, 2, 3, and 4.

A total of 108 endodontic resin blocks were utilized and these blocks were photographed at various stages, encompassing preoperative, intraoperative, and postoperative phases. The high-quality images were acquired using a digital camera Olympus E500 (Olympus, Tokyo, Japan) equipped with a macro lens, employing a shutter speed of 1.6 s and aperture f/22. To ensure standardized imaging, a reproduction table with a fixed focal length and a millimetre base was used to position the blocks consistently (Figure 1A). In the manual instrumentation groups, a series of three sets of photographs were taken, which were preoperative, intraoperative (captured after mid-coronal instrumentation with a Gates–Glidden bur), and postoperative images. In the PTG system groups, three images were also documented: preoperative, intraoperative (following the S2 instrument use), and postoperative pictures. Conversely, in the WOG system groups, only two images were recorded: preoperative and postoperative images. 

**Figure 2:**
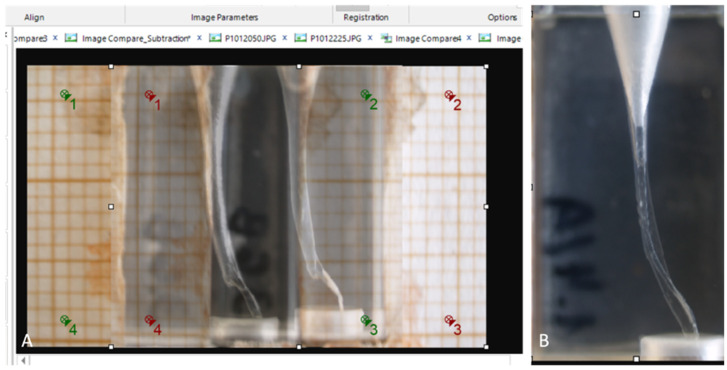
(**A**) Points registrations marked 1–4 for adequate alignment of images (in green is source point in image 1 and red is destination point in image 2 and (**B**) the overlay and comparison of images.

The obtained images were subsequently analysed using the Image-Pro 10 software (Media Cybernetics, Rockville, MD, USA) by an operator (KB). The software was calibrated to convert pixel-based measurements into millimetres and configured to extract specific measurements from the images. The grid overlay tool was applied to the initial image (Figure 1B), allowing designated points for measurement. To assess the extent of resin removal, three specific points were identified. The first point, denoting apical deviation, was located precisely 1.0 mm below the working length, the second point was positioned at the second curvature, leading to two measurements: the minimum and maximum resin removal and the third point was situated above the first, representing the average wear at the cervical region (Figure 1C). To measure the degree of resin removal, the images were analysed using the image compare tool. The points registration tool ensured accurate alignment by marking four source points in green from image 1 and four destination points in red from image 2 (Figure 2A). This enabled the image compare tool to generate a new image with preconfigured measurements (Figure 2B).

After the technical procedures, the students were requested to complete a custom-made validated questionnaire (Supplement File). Each student answered three identical questionnaires on different days, regardless of the technique they had used that day. The questionnaires were completed autonomously and anonymously, with only the day and the student’s group being identified. The questionnaire had five multiple-choice questions aimed at assessing the students’ perceptions of ease or difficulty in the various preparation stages, number of files used, and their overall satisfaction with the technique employed. This survey aimed to capture the students’ subjective experiences and impressions of the techniques during this study.

### 2.2. Statistical Analysis

The statistical package for social sciences (SPSS) software version 24 (IBM Corp., Armonk, NY, USA) was used to analyse the recorded data statistically. To analyse and compare dependent variables (apical deviation, amount of resin removed, mid cervical wear, and time) based on the instrumentation technique, the Friedman test, along with multiple comparisons using the Wilcoxon test and Bonferroni correction, was applied. For assessing instrument and technique satisfaction across the groups, the Kruskal–Wallis test and multiple comparisons with the Mann–Whitney U test were utilised. A significance level of *p* < 0.05 was established for this study.

## 3. Results

The analysis revealed significant differences among the three instrumentation techniques in terms of changes in canal conformation. Apical deviation (AD) was significantly lower in the PTG (0.073 ± 0.003) compared to the SS (0.085 ± 0.005; *p* < 0.001) and WOG (0.081 ± 0.003; *p* < 0.001). For the minimum amount of resin removed (QMin), the SS instrumentation displayed both a significantly lower average value (0.259) and a more widespread distribution compared to PTG (0.3) and WOG (0.299) (*p* < 0.001). The maximum amount of resin removed (QMax) exhibited a similar trend to QMin. The SS instrumentation yielded a significantly lower average value (0.521) compared to PTG (0.574) and WOG (0.572) (*p* < 0.001), with a wider distribution of values. Mid-cervical wear (MCW) showed statistically significant differences only between PTG and the SS instrumentation (*p* < 0.05) and between PTG and WOG (*p* < 0.001), all presented in Table 1. 

The instrumentation times also varied significantly among the techniques. Instrumentation with SS was the most time-consuming and displayed more variability in time, taking an average of 33 min and 15 s (±08:43), significantly longer than PTG (06:27 ± 02:11; *p* < 0.001) and WOG (06:34 ± 02:21; *p* < 0.001), as shown in Table 2.

Student preferences varied among instrumentation techniques, as confirmed by the Cohen’s kappa values, which confirmed no agreement between any of the instrumentation techniques and the variables relating to the instrumentation process and technique (zone, difficulty, number of files, and satisfaction). The apical third posed the greatest challenge, and the shaping phase was the most common for SS and WOG, while PTG favoured the finishing phase. PTG received the highest satisfaction ratings, followed by WOG, with SS ranked the lowest. Over 90% of operators recommended mechanical instrumentation techniques, while only 61.1% endorsed SS; thus, statistically significant differences were found between the manual technique and the mechanical techniques (*p* < 0.01 for SS vs. PTG; *p* < 0.001 for SS vs. WOG). Notably, the number of files was deemed excessive in the SS instrumentation by 47.2% of operators, whereas only 2.8% held this view for PTG or WOG. Most operators considered the file count in mechanical techniques sufficient. However, 13.9% found WOG’s file count insufficient, in contrast to the PTG system, which had no such concerns. No significant differences were found between the two mechanical instrumentation techniques (Table 3 and Table 4). 

## 4. Discussion

The various stages of endodontic treatment are closely interconnected, with each stage playing a critical role in the overall success of the treatment. Among these, biomechanical preparation—encompassing both mechanical and chemical preparation—is particularly crucial. Biomechanical preparation involves the use of endodontic instruments, files, and irrigating solutions to create optimal conditions for sealing the root canal space [17]. The advent of NiTi instruments has significantly facilitated mechanical preparation of the root canal [18]. Its importance is underscored by numerous studies that examine instruments and their properties [19,20,21,22], instrumentation techniques and movement types [22,23], and the design of the final preparation shape [5,14,24,25].

From the results obtained in the current study, the null hypothesis that there are no differences in the instrumentation techniques used regarding the quality of preparation, timing, and satisfaction is therefore rejected. One of the variables assessed is the quality of canal preparation on resin blocks performed by the students. In assessing the changes in the original design of the endodontic blocks after instrumentation, we found that the maximum and minimum resin removal showed similar results. Both indicated significantly lower values (*p* < 0.001) for the SS instrumentation when compared to the others. This could be due to the SS instruments’ design, which inherently produces a more cylindrical profile because of its 2% taper. Also, the significant differences observed between PTG and SS, as well as between PTG and WOG, aligns with previous studies showing that the PTG system excels in preserving the canal’s original shape with minimal apical deviation, as recorded in this study, but has been associated with increased debris extrusion and reduced cutting action, as shown in several studies [25,26,27,28,29]. The lower apical deviation observed in this study contrasts with the findings of Algar et al. [30], who reported a higher degree of apical transportation with the PTG system. This difference may be attributed to the comparison in their study, as the PTG system was evaluated against a system with a lesser taper percentage, which provides greater flexibility in the file. Whilst the WOG system has also been reported to preserve the initial canal shape, it tends to induce greater wear on the dentine surface [31], which could explain the increased cervical wear that was found in both the WOG and SS instrumentation groups, as the larger cervical diameter of the WOG instruments causes more cutting effect to be applied to the cervical region of the endodontic block during instrumentation, while that of the SS instrumentation group could be attributed to the use of Gates–Glidden (GG) drills, which are known for their active cutting activity in the coronal and middle thirds of the canal.

Although the learning sequence for each of the six groups was not considered as a variable for assessment, an analysis was conducted to explore the impact of the sequence of execution in different techniques. The outcome mirrored those observed when examining variables in isolation, where statistical differences were found within the manual instrumentation with the SS file group for apical deviation, minimum resin removal, and wear in the middle-cervical third, implying that the learning sequence or the order of instrumentation technique does not significantly affect the quality of preparations, which is in agreement with a previous study by Sonntag et al. [32], where a prior experience in any of the technique had no influence on the other. Specifically, in terms of apical deviation, the PTG system outperformed the SS and WOG instrumentation techniques, significantly aligning with studies indicating that the PTG system minimizes alterations to the original apical foramen position [25]. In terms of differences in instrumentation time between the techniques, the SS method took an average of 33 min and 15 s, a duration roughly five times longer than that of ProTaper Gold and WaveOne Gold. The observed time-efficiency of mechanical endodontic preparations supported by previous studies [12,13,14,15,33] reduces the overall duration of endodontic treatments, but also holds the potential to decrease the number of visits required for completion [28]. In accordance with this, both mechanical and NiTi-based systems offer a short learning curve, enabling even inexperienced operators to prepare simulated canals consistently and predictably within a shorter timeframe [15,28,34], without the apparent necessity for prior manual technique instruction.

In the final phase of this study, students were surveyed to gather their perceptions of each instrumentation technique. Perceptions indicated variations in technique evaluation, with the apical third consistently considered the most challenging, but differing views on the easiest stage. When students used SS files and the WOG system, “shaping” was regarded as the easiest, whereas with the PTG system, the “finishing” phase was favoured. Notably, a significant difference in opinions about the number of files emerged between manual and mechanical techniques. Approximately 47.2% of operators perceived SS instrumentation necessitated a substantial number of files and the majority found that the number of files used in mechanical techniques was adequate, which was not the case with manual stainless steel files. Operator satisfaction differed significantly between mechanical and manual methods, with the PTG and WOG systems being favoured. Over 90% of the students preferred mechanical instrumentation, and this pattern persisted when evaluating satisfaction concerning group/learning sequence and instrumentation technique, which is in agreement with previous studies [10,32,35,36]. The consistent feedback implies that the greater satisfaction with the PTG, followed by WOG, and lastly, the SS, can be attributed to the reduced preparation times, enhanced ease of use, and improved design made possible through mechanical instrumentation. The insights and feedback from students play a pivotal role in continually improving the curriculum and fostering stronger teacher–student relationships [37]. This role is especially significant in the context of refining challenging subjects, such as endodontics, where intricate anatomical variations add to the perceived complexity [38]. Hence, the importance of nurturing self-confidence and integrating new techniques into undergraduate education, especially through hands-on laboratory training, cannot be overstated, as it lays the foundation for lifelong learning [39]. This study offers insights into the capabilities of budding clinicians when introduced to advanced mechanical techniques and highlights the potential benefits of their incorporation into undergraduate dental curricula.

While the introduction of the NiTi alloy and subsequent mechanical preparation of the root canal has undeniably marked a significant milestone in the field of endodontics [19], it is essential to acknowledge that the ongoing introduction of various techniques aimed at simplifying root canal preparation continues to present challenges for both clinicians and students [10]. The current study, however, focused on comparing three different techniques for preparing the root canal system, using resin blocks designed for endodontic training. These blocks feature S-shaped canals that emulate root canal anatomy without its intricacies, which are often a primary cause of blockages and instrument fracture. It is worth noting that these simulated canals possess the required level of technical complexity as per the European Society of Endodontology (ESE) standards [7]. These simulated canals may exhibit qualitative differences compared to natural teeth, but they remain a valuable tool for in vitro studies, facilitating standardized comparisons between different preparation methods [40,41].

The limitations in this study that require consideration include the evaluation method for images, utilizing the ImagePro software. Despite the presence of control features for image overlapping, this method retains subjectivity linked to the operator’s actions. In order to limit the influence of the operator and reduce bias, only a single operator assessed the images without identifiable factors depicting which group the images to be assessed belong to. The grouping and numbering were completed by a different operator who had no knowledge of when the assessment and measurements were to be conducted by the other blinded operator. Also, the current study did not assess technical errors such as elbows, ledges, zipping, and instrument breakage that could impact the overall quality of the instrumentation and timing. Additionally, the administered questionnaires given to students lacking prior training could potentially affect response accuracy. Finally, the finding that continuous rotation outperformed reciprocating motion is based on the parameters assessed and feedback from students without prior experience. Therefore, caution is advised in interpreting these results, as outcomes may vary with other student cohorts. To address these concerns, future research should explore alternative assessment techniques and involve students with practical and clinical knowledge in understanding and comparing the influence of prior exposure and training on the perception of techniques and preference of instrument.

## 5. Conclusions

Within the constraints of this study, the mechanical instrumentation performed by undergraduate dental students with no prior experience was faster and with reduced apical deviation. The mechanical instrumentation outperformed the manual instrumentation in terms of root canal preparation quality. These findings underscore the potential advantages of integrating mechanical instrumentation techniques into undergraduate endodontic curricula.

## Figures and Tables

**Table 1:** Comparison of the dependent variables by instrumentation technique.

	Instrument and Technique							
	SS		PTG		WOG			
	$\bar{x}$ (SD)	[Min, Max]	$\bar{x}$ (SD)	[Min, Max]	$\bar{x}$ (SD)	[Min, Max]	*p*	MC
AD (mm)	0.085 (0.005)	[0.005, 0.076]	0.073 (0.003)	[0.067, 0.078]	0.081 (0.003)	[0.074, 0.088]	<0.001	SS-PTG *** PG-WOG ***
QMin (mm)	0.259 (0.032)	[0.032, 0.196]	0.3 (0.005)	[0.287, 0.308]	0.299 (0.004)	[0.293, 0.305]	<0.001	SS-PTG *** SS-WOG ***
QMax (mm)	0.521 (0.047)	[0.047, 0.401]	0.574 (0.003)	[0.568, 0.581]	0.572 (0.004)	[0.565, 0.579]	<0.001	M-PTG *** SS-WOG ***
MCW (mm)	0.449 (0.058)	[0.058, 0.377]	0.492 (0.002)	[0.488, 0.498]	0.485 (0.003)	[0.479, 0.491]	<0.001	SS-PTG * PTG-WOG ***

Multiple comparisons, MC, using the Wilcoxon test and Bonferroni correction with only significant comparisons shown in the MC column. SS: Stainless steel file; PTG: ProTaper Gold; WOG: WaveOne Gold; $\bar{x}$: sample mean; SD: sample standard deviation; min: minimum; max: maximum; AD: apical deviation; Qmin: minimum resin removed; QMax: maximum resin removed; MCW: mid-cervical wear; * *p* < 0.05; *** *p* < 0.001

**Table 2:** Comparison of elapsed time by instrumentation technique.

	Instrument and Technique							
	SS		PTG		WOG			
	$\bar{x}$ (SD)	[Min, Max]	$\bar{x}$ (SD)	[Min, Max]	$\bar{x}$ (SD)	[Min, Max]	*p*	MC
Time (m:s)	33:15 (08:43)	[19:37; 55:43]	06:27 (02:11)	[02:39; 14:36]	06:34 (02:21)	[03:22; 13:00]	<0.001	SS-PTG *** SS-WOG ***

SS: Stainless steel file; PTG: ProTaper Gold; WOG: WaveOne Gold; $\bar{x}$: sample mean; SD: sample standard deviation; min: minimum; max: maximum; m: minutes; s: seconds; *** *p* < 0.001.

**Table 3:** Descriptive statistics and comparisons of the variables collected through the questionnaire by instrumentation technique.

		Instrument and Technique						
		SS	PTG	WOG	V	Cohen’s *κ*		
		n (%)	n (%)	n (%)		SS vs. PTG	SS vs. WOG	PTG vs. WOG
Difficult zone	None	0 (0%)	12 (33.3%)	11 (30.6%)	0.341 ***	0.039	0.129	0.261
	Cervical	2 (5.6%)	0 (0%)	0 (0%)				
	Middle	9 (25%)	1 (2.8%)	2 (5.6%)				
	Apical	25 (69.4%)	23 (63.9%)	23 (63.9%)				
Easiest phase	None	0 (0%)	1 (2.8%)	2 (5.6%)	0.282 **	0.123	−0.129	−0.050
	Shaping	23 (63.9%)	7 (19.4%)	15 (41.7%)				
	Finishing	8 (22.2%)	17 (47.2%)	9 (25%)				
	Shaping and finishing	5 (13.9%)	11 (30.6%)	10 (27.8%)				
Number of files	Insufficient	1 (2.8%)	0 (0%)	5 (13.9%)	0.427 ***	0.057	0.019	0.118
	Sufficient	18 (50%)	35 (97.2%)	30 (83.3%)				
	Excessive	17 (47.2%)	1 (2.8%)	1 (2.8%)				
Technical satisfaction	1	3 (8.3%)	0 (0%)	1 (2.8%)	0.540 ***	0.025	−0.041	0.118
	2	13 (36.1%)	0 (0%)	2 (5.6%)				
	3	11 (30.6%)	0 (0%)	3 (8.3%)				
	4	8 (22.2%)	6 (16.7%)	9 (25%)				
	5	1 (2.8%)	30 (83.3%)	21 (58.3%)				

(Cramer’s V and multiple comparisons (MC) using Cohen’s kappa coefficient of agreement, κ). SS: stainless steel file; PTG: ProTaper Gold; WOG: WaveOne Gold; n: absolute frequency; %: relative frequency; ** *p* < 0.01; *** *p* < 0.001.

**Table 4:** Descriptive statistics and comparisons of technique satisfaction.

		Instrument and Technique							
		SS		PTG		WOG		*p* ^B^	MC
		$\bar{x}$ (SD)	[Min; Max]	$\bar{x}$ (SD)	[Min; Max]	$\bar{x}$ (SD)	[Min, Max]		
Group	I	2.8 (0.8)	[2, 4]	5.0 (0)	[5, 5]	4.7 (0.5)	[4, 5]	0.006	SS-PTG *
	II	2.3 (1.2)	[1, 4]	5.0 (0)	[5, 5]	5.0 (0)	[5, 5]	0.002	SS-PTG * SS-WOG *
	III	3.2 (1.5)	[1, 5]	5.0 (0)	[5, 5]	5.0 (0)	[5, 5]	0.007	-
	IV	3.5 (0.8)	[2, 4]	4.3 (0.5)	[4, 5]	3.2 (1.2)	[2, 5]	0.157	-
	V	2.2 (0.4)	[2, 3]	5.0 (0)	[5, 5]	4.7 (0.5)	[4, 5]	0.004	SS-PTG *
	VI	2.5 (0.5)	[2, 3]	4.7 (0.5)	[4, 5]	3.3 (1.2)	[1, 4]	0.006	SS-PTG **
*p* ^A^		0.149		0.006					
MC				IV-I * IV-II * IV-III * IV-V *		VI-II * VI-III * IV-II * IV-III *			

*p* ^A^: significance of each instrument or technique among the six groups. *p* ^B^: significance of individual group among the three instruments and techniques. (Kruskal–Wallis’s test and multiple comparisons (MC) with Mann–Whitney U test and Bonferroni correction.) Only significant comparisons are indicated in the MC column/row. SS: stainless steel file; PTG: ProTaper Gold; WOG: WaveOne Gold; $\bar{x}$: sample mean; SD: sample standard deviation; min: minimum; max: maximum. * *p* < 0.05; ** *p* < 0.01. Group I: SS-PTG-WOG; Group II: SS-WOG-PTG; Group III: PTG-SS-WOG; Group IV: PTG-WOG-SS; Group V: WOG-SS-PTG; Group VI: WOG-PTG-SS.

## Data Availability

The datasets used and/or analysed in the current study are available from the corresponding author upon reasonable request.

## Supplementary Materials

The following supporting information can be downloaded at: https://www.mdpi.com/article/10.3390/dj12110363/s1.
