# Supplementary material for: Evaluation and Comparison of Manual and Mechanical Endodontic Instrumentation Completed by Undergraduate Dental Students on Endodontic Blocks
Source: Dent J (Basel). 2024 Nov 14;12(11):363. doi: 10.3390/dj12110363 (PMC11592560; doi:10.3390/dj12110363)
Supplement: Supplementary file 1 [file dentistry-12-00363-s001.zip › dentistry-3276115-supplementary.pdf]

Date: ____/______/2020

Group: ( ) 1 ( ) 2 ( ) 3 ( ) 4 ( ) 5 ( ) 6

QUESTIONNAIRE

This questionnaire aims to assess each student's experience and individual perception regarding the use of different instrumentation techniques in acrylic test blocks. Please mark with an (X) the answer(s) that you find most appropriate.

1) Which canal thirds did you find most difficult to prepare?

( ) cervical

( ) middle

( ) apical

( ) none of the above

2) Which phase of preparation was easiest to perform?

( ) Shaping – beginning

( ) Finishing – end

( ) Shaping and Finishing

( ) none of the above

3) What do you think about the number of files you needed to use today?

( ) Sufficient

( ) Insufficient

( ) Too many

4) What is your satisfaction level with the technique (system) you used today? Where 0 (zero) corresponds to very dissatisfied and 5 (five) to extremely satisfied.

( ) 0 ( ) 1 ( ) 2 ( ) 3 ( ) 4 ( ) 5

5) Would you recommend using this technique (system) to other students?

( ) Yes ( ) No
